# The treatment of medial tibial stress syndrome in athletes; a randomized clinical trial

**DOI:** 10.1186/1758-2555-4-12

**Published:** 2012-03-30

**Authors:** Maarten Hendrik Moen, Leonoor Holtslag, Eric Bakker, Carl Barten, Adam Weir, Johannes L Tol, Frank Backx

**Affiliations:** 1 Rehabilitation and Sports Medicine Department, University Medical Center Utrecht, Utrecht, Holland; 2 Department of Orthopedic Surgery, Academic Medical Center Amsterdam, Amsterdam, Holland; 3 Department of Clinical Epidemiology, Biostatistics and Bioinformatics, Academic Medical Center Amsterdam, Amsterdam, Holland; 4 Department of Physical Therapy, Academy of Physical Education, The Hague, Holland; 5 Department of Sports Medicine, Medical Center Haaglanden, Leidschendam, Holland

**Keywords:** Running program, Exercises, Compression sleeve, Shin splints

## Abstract

**Background:**

The only three randomized trials on the treatment of MTSS were all performed in military populations. The treatment options investigated in this study were not previously examined in athletes. This study investigated if functional outcome of three common treatment options for medial tibial stress syndrome (MTSS) in athletes in a non-military setting was the same.

**Methods:**

The study design was randomized and multi-centered. Physical therapists and sports physicians referred athletes with MTSS to the hospital for inclusion. 81 athletes were assessed for eligibility of which 74 athletes were included and randomized to three treatment groups. Group one performed a graded running program, group two performed a graded running program with additional stretching and strengthening exercises for the calves, while group three performed a graded running program with an additional sports compression stocking. The primary outcome measure was: time to complete a running program (able to run 18 minutes with high intensity) and secondary outcome was: general satisfaction with treatment.

**Results:**

74 Athletes were randomized and included of which 14 did not complete the study due a lack of progress (18.9%). The data was analyzed on an intention-to-treat basis. Time to complete a running program and general satisfaction with the treatment were not significantly different between the three treatment groups.

**Conclusion:**

This was the first randomized trial on the treatment of MTSS in athletes in a non-military setting. No differences were found between the groups for the time to complete a running program.

**Trial registration:**

CCMO; NL23471.098.08

## Introduction

One of the most common causes of overuse leg injuries is medial tibial stress syndrome (MTSS) with incidences varying between 4 and 35% in athletic and military populations [1-3]. In the past the etiology of this syndrome was not clear, and several possible causes were described e.g. increased intracompartimental pressure or a traction induced periostitis [4,5]. Recently, different imaging techniques have demonstrated that the tibial cortex is **probably **involved in MTSS. With dual energy x-ray absorptiometry (DEXA) scanning Magnusson et al. showed that decreased bone density was present in the symptomatic part of the tibia [6]. High resolution computer tomography (CT) scans revealed osteopenia in the involved tibial cortex [7]. **However, histological studies are needed in which the bone overload theory is confirmed. Until then, bone overload as a cause of MTSS remains a hypothesis**.

Despite the high incidence of MTSS, a recent systematic review of the literature only identified three randomized controlled trials in the treatment of MTSS, all performed in military population [8]. In the study by Andrish et al. five different interventions (ice application, aspirin and ice application, phenylbutazone and ice application, heel cord stretching and ice application, walking cast) were compared. The outcome measure was being able to run 500 meters comfortably [1]. The study by Nissen et al. studied if the application of gallium-arsenic laser treatment compared to sham laser treatment shortened the time to return to duty [9]. Johnston et al. investigated if a leg brace added to a rehabilitation program influenced the time to complete 800 meters of running pain free [10]. None of these studies found that the intervention group recovered significantly faster than the control group [1,9,10]. Besides these three RCTs there were two non-randomized controlled studies [11,12], and a few lower quality studies found [13-17]. In these treatment studies many different outcome measures were used. There is no recognized validated outcome measure for MTSS.

In clinical practice, graded running, strengthening and stretching exercises for the calf muscles are frequently prescribed for MTSS [18,19]. Graded running in itself could strengthen the tibial cortex [20-22]. Waldorff et al. showed that physiological loading allowed increased remodeling of the tibiae and increased resorption of micro-damage [22]. While very few studies have been published on the effect of stretching for MTSS [1,12], stretching is frequently included in treatment programs. Some research has been published on the effect of muscles in protecting the cortex. Animal and human studies showed that diminished muscle force negatively influences the bone adaptation process. Weaker muscles that oppose tibial bending allow an increase in bending to occur [23-26]. A recent military study showed that tibial strain, measured with strain gauges, increased after performing fatiguing long distance marches [27].

Sports compression stockings are used frequently in the Netherlands to treat MTSS [28]. A sports compression stocking might provide direct compression of the tibia and via the surrounding soft tissues, especially during intermittent loading. Compression of bony tissue has been shown to promote the expression of bone specific genes [29].

The effects of these interventions have not been previously studied in randomized trials in an athletic population. The aim of this study was to study, in a non-military, athletic population, a graded running program alone or with additional strengthening and stretching exercises or while wearing a sport compression stocking for the leg for the treatment of MTSS in a randomized trial.

## Methods

### Subjects

The design of the study was a randomized multi-center trial with three groups. Each athlete was randomly assigned to a treatment group, and all the athletes in the group received an intervention. The multi-center study was announced to physical therapists, general practitioners, sports medicine physicians and orthopedic surgeons. They informed the athletes about the existence of the study. They could all refer an athlete to a sports physician in one of three participating sports medicine clinics in the Netherlands (two large district and one university hospital). The sports physicians examined the athlete for complaints of MTSS and for suitability for inclusion. If the athlete was suitable for inclusion, the sports physician referred the athlete to one of the investigators for intake. A single sports physician identically trained the investigators for the study. The diagnosis of MTSS was made according to the criteria of Yates et al. (see Table 1) [30]. For exclusion criteria the description of symptoms provided by Edwards et al. in their recent review were used to specify stress fractures of the tibia and chronic exertional compartment syndrome (CECS) [31]. Pain in stress fractures is often focal (clinically and with physical examination) and the start of complaints is usually abrupt. Pain initially occurs as a mild ache after exercise, but as the condition progresses pain can be felt early after starting exercise. Athletes with CECS often complain of burning, cramping or pain over the involved compartment with exercise. Pain is progressive with continued exercise and will disappear after cessation of activities [31].

**Table 1:** Inclusion and exclusion criteria

Inclusion criteria	Exclusion criteria
Pain induced by exercise and present during or after exercise	Tibial fracture in the past
Pain on the postero-medial border of the tibia	History of paresthesia
Diffuse pain on palpation of the postero-medial tibia **for at least 5 centimeters**	Clinical suspicion of a tibial stress fracture [31]**or stress fracture present on x-ray**
Age > 16 years old	Clinical suspicion of exercise induced compartment syndrome [31]**or increased intra-compartimental pressure**
Active in sport at least once per week	
Complaints for more than 3 weeks	

The athletes had to be involved in sport at least once a week. The inclusion was definitive when the diagnosis MTSS by an instructed sports physician was confirmed according to the Yates et al. criteria [30] and the presence of exclusion criteria [31] was excluded (Table 1) and informed consent was given.

### Randomization

For the randomization at each location there were three identical opaque blank envelopes in a box each containing a letter, explaining to which of the three groups the athlete had been allocated. After the athlete had been allocated the letter was returned to the envelope and into the box to be used again by the next athlete.

### Intake

At baseline the investigators noted sex, weight, height, body mass index (BMI), kind of sport in which the athlete was involved, centimeters of pain on palpation of the postero-medial border of the tibia, side of the complaints and number of days with complaints. Subsequently, a running test was performed. The running test consisted of running on a treadmill at a fixed speed, while wearing the athlete's own running shoes. Although the running test is not validated for the use in MTSS athletes it has been used previously in a treatment studies on MTSS [32,33].

First, the athlete was shown a visual analogue scale (VAS) for pain by the investigators. Then the athlete was told that when a four (on a 1-10 VAS scale) for MTSS was experienced, defined as an indication that the pain was starting to become annoying, the running test had to be stopped. The running test started at 7,5 km/hour for two minutes. After this initial warming-up phase the distance was noted that could be run at 10 km/hour until a four on the VAS scale was noted. The distance ran at 7,5 km/hour was subtracted from the total meters run and was called "meters run at 10 km/h".

### Graded running program

With the result of the running test the athlete was placed in one of the six phases of the graded running program (see Table 2) [32,33]. When "meters run at 10 km/hour" was between 0-400 meters, the athlete started the running program in phase one. When 401-800 meters could be run, the athlete started in phase two. When 801-1200 meters could be run the athlete started in phase three. When 1201-1600 meters could be run, the athlete started phase four. When 1600 meters or more could be run, athletes started phase five. When pain was present already during walking no running test was performed. Then the athlete was advised about how to avoid complaints by reducing loading of the leg. When in these athletes pain was not present during walking for two consecutive days, phase one of the running program was started. The running program was performed three times per week, with a day off between each session.

**Table 2:** Running program

Running phase	Surface	Minutes	Total	Speed/intensity
**1**	Treadmill	2 **2 **2 **2 **2 **2 **2 **2**	16 minutes	2 = running at 10 km/hour, **2 **= walking at 6 km/hour
**2**	Treadmill	2 **2 **2 **2 **2 **2 **2 **2**	16 minutes	2 = running at 12 km/hour, **2 **= walking at 6 km/hour
**3**	Concrete	3 **2 **3 **2 **3 **2 **3 **2**	20 minutes	Intensity 1-2 (*)3 = running, **2 **= walking
**4**	Concrete	3 **2 **3 **2 **3 **2 **3 **2**	20 minutes	Intensity 2-3 (*)3 = running, **2 **= walking
**5**	Concrete	Continuous running	16 minutes	Intensity 1-2 (*)
**6**	Concrete	Continuous running	18 minutes	Intensity 2-3 (*)

(*): Intensity 1; running speed: light jogging. Intensity 2; running speed; jogging while able to speak. Intensity 3; running speed: jogging while speaking becomes difficult

A new phase of the running program could be commenced if a phase was finished without a pain score of four or higher on the 1-10 VAS pain scale during the running. When pain (four or more on the VAS scale) was present immediately after the running or the day after the running the program did not progress and running remained in the same phase and the time run was decreased by two minutes.

### Graded running program with exercises

In addition to the graded running, which is described above, athletes performed exercises at home five times per week (see addendum). The exercises consisted of stretching and strengthening exercises of the calves. The investigators practiced the exercises with the athletes until they were familiar enough to perform them at home. The exercise schedule consisted of five phases. When a certain phase could be performed without a four on a 0-10 VAS pain scale, the following phase could be started. When phase five was finished, the athlete kept on exercising with a random mix of exercises from different phases.

### Graded running program with a sports compression stocking

In addition to the graded running program, which is described above, a sports compression stocking for the leg (Herzog Medical, Woudenberg, the Netherlands) was worn when the athlete was walking or running. The compression stocking could only be taken off when the athlete was seated or laying down for more than 15 minutes. To supply the right size, the investigators measured the circumference of the calf just below the knee fold, the maximal calf circumference and the circumference just above the malleoli. Based on these measurements a size 1-6 of the stockings was supplied.

### Follow-up

Follow-up took place at week 2,4,6,8,10,12,16,22,28,34,42,50. To structure and perform follow-up, the investigators were identically trained by one sports physician (MM). Athletes were asked about progress with the running schedule, complaints and compliance with the treatment. Additionally, a physical examination was performed and feedback was provided.

### Compliance

A commonly used method to measure compliance is self-reported adherence to the treatment [34,35]. At follow-up athletes were asked to choose from the following sentences: "I stuck to the prescribed activities", "most of the time I stuck to the prescribed activities", "I stuck to the prescribed activities at the beginning, but not anymore", or, "I did not follow the prescription at all" (adapted from Kallings et al., 2009) [34]. The athletes that answered "I stuck to the prescribed activities" and "most of the time I stuck to the prescribed activities", when asked about the adherence of the prescribed activities, formed the group that adhered.

### Blinding

The athletes and investigators were not blinded. The data analyst (EB) was blinded to the chosen therapy. The athlete was not blinded to the treatment, because it was not possible to perform blinding. The investigators were not blinded, because the investigators had to give feed-back to the athlete about the intervention. The investigators also asked about compliance of the prescribed treatment.

### Outcome measurement

Primary outcome: the number of days from inclusion to the completion of phase six (being able to run 18 consecutive minutes outdoors at a speed in which speech becomes difficult) of the running schedule was used as primary outcome measurement. Although this outcome measurement was used in previous studies on the treatment of MTSS [32,33], this outcome measurement has not been validated. Unfortunately, no validated outcome measurements for MTSS exist.

When an athlete was not able to finish the running program and quit the study, the Likert scale was used to assess the status of the athlete [36]. This scale was scored as: 1 = completely recovered, 2 = much improved, 3 = somewhat improved, 4 = same, 5 = worse and 6 = much worse. When a athlete did not have progress anymore and wanted to quit the study the Likert score was collected. The Likert scale was shown by the investigator and the investigator asked how the athlete was doing at the moment of quitting the study compared to baseline. The athlete chose a number. Satisfaction with the treatment in general on a 1-10 scale was used as secondary outcome measurement, in which 1 = very dissatisfied with treatment and 10 = highly satisfied with the treatment in general.

### Data analysis

Data was entered using SPSS 17.0 (SPSS Inc, Chicago, Illinois, USA). To compare the outcome between groups Analysis of Variance (ANOVA) with post-hoc analysis according to Games-Howell was used. For dichotomized variables Chi-Square analysis was used. The athletes were analyzed by intention-to-treat. For athletes that were lost to follow-up a worst and best case scenario was calculated. For athletes that withdrew from the study due to a lack of progress, the time to complete the running program was entered as missing data in the database. Kaplan-Meier analysis was used to obtain reversed survival curves.

The local medical ethical committee agreed with the study beforehand (reference number for the study; NL23471.098.08). The committee agreed to include athletes who were 16 years of age and older. Informed consent was received from each participant.

Power analysis Previous studies on the treatment of MTSS reported a maximum time to recovery of 17.2 days and a standard deviation of 9,5 days [1,9,10]. Based on these findings, we considered a reduction of 50% in time to recovery would be clinically relevant. Sample size calculation indicated that 22 athletes (including an expected 10% lost to follow-up) per treatment group were needed to detect such difference with a power of 80% at a significance level of 0.05.

## Results

**Figure 1:**
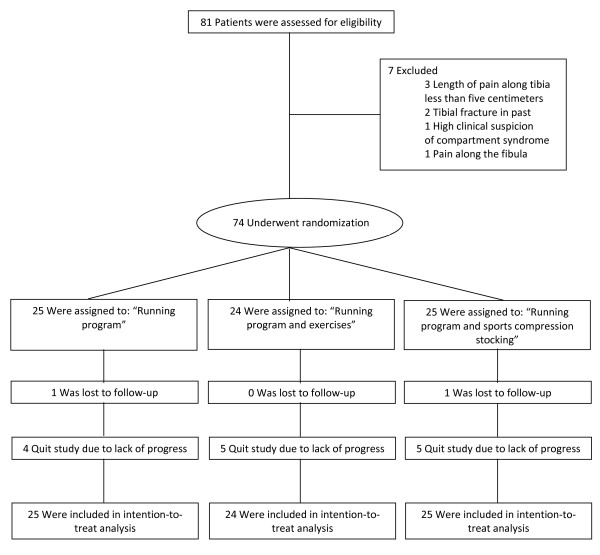
**Flow of patients through the study**.

Between October 2008 and February 2010 athletes were included in the study. 81 athletes were assessed for suitability for inclusion and 74 fitted the criteria and were randomized. The flow of athletes through the study is shown in Figure 1. The baseline characteristics for all athletes groups are presented in Table 3. No significant differences in baseline characteristics were found between the treatment groups. Most athletes (69%) started in phase 1 or 2 of the running program. No significant differences were found for the starting phase between the groups. The follow-up period ended in June 2010. The athletes were involved in different kind of sports. The most prevalent were soccer (24%), running (15%) and field hockey (10%). The mean number of hours that the athletes were involved in sport was 5,1 (SD 3,2) hours/week (range 1-21 hours/week). No significant differences in hours/week involvement in sport were found between the groups.

**Table 3:** Baseline characteristics for the three treatment groups

	Running program (SD) N = 25	Running program + exercises (SD) N = 24	Running program + compression stocking (SD) N = 25	*p*-value
**Height (centimeters)**	175,4 (4,9)	171,6 (5,1)	177,0 (9,9)	NS
**Weight (kilograms)**	68,7 (8,1)	68,3 (7,7)	70,4 (11,2)	NS
**BMI (kilograms/(length)^2^)**	22,2 (1,8)	22,9 (2,6)	22,3 (2,6)	NS
**Age (years)**	22,2 (6,8)	20,7 (6,4)	23,0 (8,2)	NS
**Sex (percentage females)**	65,2%	72,7%	53,5%	NS
**Side with complaints (percentage bilateral complaints)**	87,0%	77,3%	96,4%	NS
**Centimeters of pain on palpation**	12,2 (4,9)	11,6 (5,1)	16,1 (8,8)	NS
**Days with complaints**	178,0 (319,2)	174,0 (274,1)	213,7 (363,8)	NS
**Meters run without pain on treadmill**	708,7 (423,9)	572,6 (419,2)	591,8 (427,2)	NS
	Phase 1: 36% Phase 2: 28% Phase 3: 24% Phase 4: 12%	Phase 1: 38% Phase 2: 33% Phase 3: 17% Phase 4: 13%	Phase 1: 44% Phase 2: 28% Phase 3: 16% Phase 4: 20%	NS

Abbreviations: *NS *not significant (*p *> 0.05)

**Figure 2:**
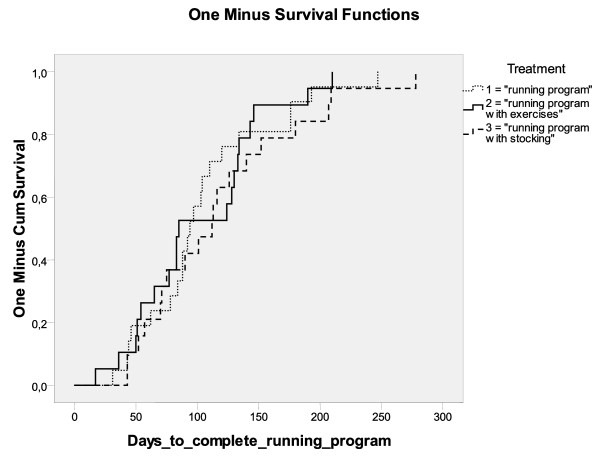
**Reversed Kaplan-Meier survival curve for days to complete the running program**.

No differences were found between the groups for primary and secondary outcome measures after intention-to-treat analysis (Table 4). The mean number of days to complete the running program was 105.2 days (SD 54.6) for the group with the running program, 117.6 days (SD 64.2) for the group with the running program and exercises and 102.1 days (SD 52.3) for the group with the running program and the sports compression stocking (*p *> 0.05). The reversed survival curve is presented in Figure 2. No significant differences were found in the number of meters able to run on quitting the study in athletes that withdrew between the groups.

**Table 4:** Primary and secondary outcome measures

	Running program (SD, 95% CI)	Running program and exercises (SD, 95% CI)	Running program and compression stocking (SD, 95% CI)	*p*-value
**Days to complete the running program**	105.2 (54.6, 80.4-130.1)	117.6 (64.2, 86.7-148.6)	102.1 (52.3, 76.9-127.2)	NS
**Satisfaction with treatment in general on 1-10 scale**	6.5 (1.3, 4,5-8,6)	5.9 (1.6, 4.6-7.3)	6.8 (2.0, 5,7-8,0)	NS

Abbreviations: *NS *not significant (*p *> 0.05), *SD *standard deviation, 95% *CI *95% confidence interval

For satisfaction with the treatment in general (secondary outcome measurement) no differences were found between the groups (*p *> 0.05). Satisfaction in the running program group was 6.5 (SD 1.3), in the running program with exercises group 5.9 (SD 1.6) and 6.8 (SD 2.0) in the running program and sports compression stocking.

No significant differences were found in the number of athletes that quit the study due to subjective lack of progress with the injury or that were lost to follow-up (see Figure 1). The Likert score for these athletes was not significantly different between the groups and ranged from 3 to 4. In a worst case/best-case scenario for the intention-to-treat analysis (the lost athletes were calculated as fastest recovery (17 days) or slowest recovery (278 days)) still no significant differences between groups could be found in days to complete the running program.

No athletes were excluded from the study due to a lack of compliance. All athletes reported, "I have stuck to the prescribed activities" or "most of the time I have stuck to the prescribed activities". No complications were reported after the treatments.

## Discussion

This is the first randomized study on the treatment of MTSS in athletes outside the military. No significant differences for time to complete a running program and athlete satisfaction were found between the treatment groups. The interventions in this study were implemented for both sexes, a wide range of different sports and ages between 16-51 years old. This means that the results from this study can be generalized to a broad athletic population. The results from this study are in keeping with the only three other published RCTs on the treatment of MTSS [1,9,10].

**Prior to the start of treatment a running test was performed, which is not validated. The running test, although not validated, was used in previous studies on MTSS **[32,33]**. The results of the running tests in these studies were more or less comparable to the findings in this study. For the future, the running test should be validated**.

In the literature no validated outcome measure for MTSS is available and therefore several outcome measures are used. The development of validated outcome measures is a priority in this research field **to increase the quality of treatment studies on MTSS**. The previous randomized studies were all conducted in a military population and used different outcome measures. Andrish et al. used no reported tenderness or being able to run 500 consecutive meters as outcome measure [1]. In the study by Nissen et al., days to return to active duty was the primary outcome measurement [9]. The study by Johnston et al. used the time to run 800 meters without pain as outcome measure [10].

This study used time to complete a running program (defined as running continuously at a pace when speech becomes difficult) as the primary outcome measure. This is similar to the studies by Andrish et al. and Johnston et al. [1,10]. In a pilot study conducted by our research group, a lot of athletes were able to run further than 800 meters during the running test at intake. That is why the decision was made to lengthen the running program compared to these studies.

No significant differences between the groups for primary and secondary outcome measures were found. **Therefore, if MTSS is treated with a running program, no large additional effect of the two interventions can be expected**. It should however, be noted that a graded running program has not been compared with a control group that rested in any study. Now, only assumptions can be made that the graded running program improves the density and strength of the tibia, and that rest does not have this effect. This is why no conclusions can be drawn from this or other studies that a graded running program is superior to rest. While setting up the study, it was tried to include a control group that rested. However, several physical therapists, sports physicians and orthopedic surgeons did not want to participate in the study if the control group rested, because they believed then they couldn't offer anything to the athletes. This was the reason that the control group performed a graded running program.

Self- reported adherence to the treatment was used to quantify compliance. This method of quantifying adherence carries a potential risk of bias, including social desirability [34]. Nevertheless, self-reported adherence has been found to be accurate and reliable when compared to objective quantification of physical activity [34,35]. No gold standard for quantifying adherence to physical activity or physical activity levels exist [37].

In all three groups athletes quit the study due to a lack of progress. These athletes were included in the analysis and this did not affect the outcome. With a relatively high dropout percentage (18,9%), this is a shortcoming of the study. The number of athletes that quit was not significantly different, with a dropout percentage varying between 16,0 and 20,8%.

Another limitation of this study is the lack of blinding of the athletes and the investigators. The studied treatment modalities were so different, that it was very hard to apply blinding to the athletes. The investigators were not blinded, as they had to give feedback to the athletes on the treatment received.

One of the weakness of this study is the power analysis used. At the start of the study, based on the available information from military studies [1,9,10], we assumed that 22 athletes per treatment group were needed to find a clinically relevant reduction of 50% in time to recovery, i.e. from 17 days to 8-9 days, with alpha set on 0.05 and a power of 0.8. However, recent studies [12,32,33] indicated that a time to recovery of 60-100 days is likely to be more realistic in athletes with MTSS. The current study was therefore able to detect a large effect of the interventions. For future studies, with the data from these studies and the data from this study a more precise power analysis could be possible [12,32,33].

## Conclusion

This is the first randomized controlled study on MTSS in athletes outside a military setting. No significant differences were found between the three treatment groups in days to complete a running program (primary outcome measure) and satisfaction with the treatment (secondary outcome measure). This study does provide insight in recovery of MTSS in athletes with an average time to complete a running program of 102.1 (SD 52.3) - 117.6 days (SD 64.2). Further RCT's should be performed to test the hypothesis that a graded running program leads to a favorable outcome compared with rest. In future studies validated outcome measures should be developed and new interventions can be tested by comparing their effectiveness to a graded running program.

## Competing interests

We declare that none of the authors has any financial or non-financial competing interests.

## Authors' contributions

MM designed the study and instructed the physical therapists, sports physicians, orthopedic surgeons and investigators. LH was one of the leading investigators and initiated patient contacts at the beginning of the study. EB performed the statistical analysis after the data was obtained. CB was one of the leading physical therapists involved in the study and had a large role in recruiting patients. AW was involved in designing the study methodologically and practically. JT was involved as well in designing the study methodologically and practically. FB coordinated the process of conducting the study. All authors read and approved the final manuscript.
